# Hematobiochemical alterations of acute chlorpyriphos intoxication in indigenous chicken

**DOI:** 10.14202/vetworld.2015.750-754

**Published:** 2015-06-20

**Authors:** Shameem Ara Begum, Tirtha Nath Upadhyaya, Gautam Kumar Baruah, Taibur Rahman, Debesh Chandra Pathak, Kabita Sarma, Rumi Saikia Bora

**Affiliations:** 1 Department of Veterinary Pathology, College of Veterinary Science, Assam Agricultural University, Khanapara, Guwahati, India; 2 Department of Anatomy and Histology, College of Veterinary Science, Assam Agricultural University, Khanapara, Guwahati, India; 3 Department of Livestock Production and Management, College of Veterinary Science, Assam Agricultural University, Khanapara, Guwahati, India

**Keywords:** acute toxicity, biochemical, chlorpyriphos, hematology

## Abstract

**Aim:**

The present investigation was undertaken to elaborate hematobiochemical alterations of acute chlorpyriphos (CPF) toxicity in indigenous chicken. Since there is no available literature on the detailed hematobiochemical changes of CPF in indigenous chicken, hence, the present study was designed to establish toxicological effect of CPF on blood biochemical parameters of indigenous chicken which are at a great risk of exposure to pesticides. These will help physiologist, pathologist, and poultry scientists for effective production strategy as well as disease control regime.

**Materials and Methods:**

The birds were divided into two major Groups I and II. Group I served as control and Group II was treated with CPF (36 mg/kg). Blood samples were assayed for hemoglobin (Hb), total erythrocyte count (TEC), total leukocyte count (TLC), differential leukocyte count, and biochemical constituents such as alkaline phosphatase (ALP), aspartate aminotransferase (AST), alanine aminotransferase (ALT), cholinesterase (CHE), total protein, and uric acid.

**Results:**

Hb, TEC, and TLC levels increased significantly (p<0.01) in toxin fed birds, whereas, lymphocyte percent decreased significantly, and heterophil percent increased significantly. Serum ALP, AST, ALT, and uric acid increased significantly in CPF treated birds. Decreased serum CHE values were observed in CPF fed group. The protein level remained almost same. Uric acid level was found to be increased significantly in the treated group compared to control.

**Conclusion:**

The results indicated that acute CPF intoxication produce changes in hematology and biochemical constituents of the treated birds.

## Introduction

Chlorpyriphos (CPF) (O, O-diethyl O-3,5,6-trichloro-2-pyridyl phosphorothioate) is one of about 100 organophosphate (OP) insecticides in the market today. It is used to kill insect pests by disrupting their nervous system. CPF has an advantage over other products in that it is effective against a wide range of plant-eating insect pests. CPF is widely used organothiophosphate pesticide for domestic and agricultural applications throughout the world. CPF induces deleterious effects primarily through acetylcholinesterase inhibition and produces symptoms characteristic of cholinergic overstimulation such as salivation, nausea, vomiting, tremor, and convulsions in mammalian species including human beings [1].

CPF is used in controlling a variety of insects, flees, termites, lice, etc. It is used as an insecticide on grain, fruit nut, and vegetable crops. Residual amounts of CPF are detected in water, soil, fabric, and on surfaces for months to years [2]. CPF is also applied to the soil surrounding or beneath buildings as protection against termites including chicken houses [3]. Chickens are more commonly affected with pesticide toxicity because poultry houses are frequently dusted with pesticides. Chemical pesticide causes health consequences to the birds culminating in great economic loss. It is also posing a potential threat to public health due to the presence of pesticide residues in poultry meat and egg. Ample evidence exists to suggest that the use of pesticides on crops, in storehouses, in poultry houses plus the nonjudicious application for spraying animals or in dipping solutions to prevent ectoparasites leaves behind its residue causing serious health effects [4].

Assam is mainly an agriculture based state, where pesticides are widely used by the farmers. The year wise consumption of pesticides including CPF in Assam was 150 MT, 143 MT, and 141 MT during the year 2008-2009, 2009-2010, and 2010-2011, respectively. According to rural veterinary field officers (unpublished data) spraying of pesticides on many occasions may cause accidental toxicity and deaths among indigenous poultry of rural areas in Assam, as these birds are let loose in the field during the daytime. There is every possibility of picking up of toxic substances from the grain and dead insects available in the grazing field. Limited information is available on CPF induced pathological changes on various organs in chicken. Reports on toxicological studies on CPF from North-Eastern region of India could not be traced out in the available literature. Therefore, the present study has been undertaken to study the ­hematobiochemical alterations of acute CPF intoxication in indigenous chicken.

## Materials and Methods

### Ethical approval

The experimental trials were approved by the Institutional Animal Ethics Committee (No.770/ac/CPCSEA/FVSc/AAU/IAEC/11-12/128), and conducted under its guidelines.

### Animals

3-month-old unsexed 24 indigenous chickens were procured from All India Coordinated Research Project on Poultry, College of Veterinary Science, AAU, Khanapara Guwahati – 781 022 were wing banded, weighed, and reared in the Department of Pathology, College of Veterinary Science, AAU, Khanapara Guwahati - 781 022 with *ad libitum* supply of feed and water. They were randomly distributed into two groups of 12 chickens each, i.e., control; CPF treated.

### Chemical (insecticide)

Commercial products of CPF (20%) used in this study was procured from Excel Crop Care Private Limited, Mumbai, India.

### Treatment groups

In this study, 24 chickens were randomly segregated into two groups of 12 each and fasted for 6 h prior to dosing. Following the period of fasting, the birds were weighed, and the doses were calculated according to the body weight. The insecticides were diluted in distilled water to obtain the desired concentrations. Fresh preparations were orally administered daily using oral gavage. The first group was given 36 mg/kg bw CPF as a single dose, the second group was given distilled water in the same route and served as control. The birds were closely watched for the presence of clinical signs if any, till death. All the treated birds died within 4-36 h of dosing. Birds of the control group were sacrificed at the end of the experiment.

### Sampling

About 2 ml of blood from the birds were aseptically collected from the jugular vein/wing vein with a sterile 2 ml disposable syringe. In the treatment group, blood was collected at 0 h and after treatment at every 2 h interval up to 6 h, then at 12 h, and subsequently at 12 h interval till death. In the control group, blood was collected at the same time and same day of collection as in the case of a treated group. About 1 ml of blood was taken in a vial containing EDTA as anticoagulant @ 1mg/ml, for estimation of hemoglobin (Hb) and total erythrocyte count (TEC) and the remaining 1 ml of blood was kept in a dry wide-mouthed test tube in slanting position at room temperature for separation of serum. The serum was separated after 6-8 h following collection and stored at −20°C for biochemical analysis.

### Hematology

Hb, TEC and total leukocyte count (TLC) were estimated with the help of automated hematology cell counter (Model-ms4e). Differential leukocyte count was analyzed as per standard method [5].

### Biochemical assays

Plasma was separated and immediately used for the analysis of acetylcholine esterase enzyme (CHE), aspartate aminotransferase (AST), alanine aminotransferase (ALT), alkaline phosphatase (ALP), uric acid, total protein, and albumin. Biochemical tests were performed using commercial kits (Siemens Diagnostics India Ltd) on an Ultraviolet-visible spectrophotometer.

### Statistical analysis

All data were expressed as mean ± standard error. The statistical significance of the mean differences between control and treated groups was analyzed by one-way ANOVA. Statistical calculations were performed with the SPSS 11.5 computer program (SPSS Inc. Chicago, Illinois, USA). The values of (p<0.05) and (p<0.01) were taken as the cut-off value to consider differences statistically significant and highly significant, respectively.

## Result and Discussion

**Figure-1:**
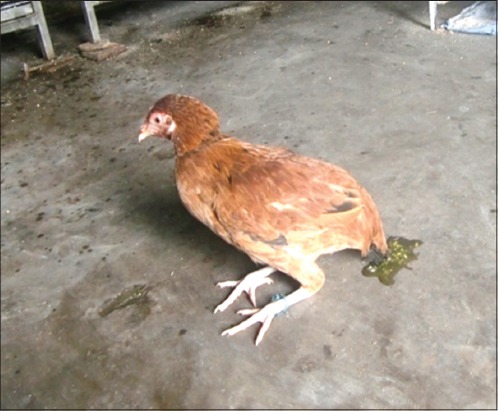
Chicken showing diarrhea.

**Figure-2:**
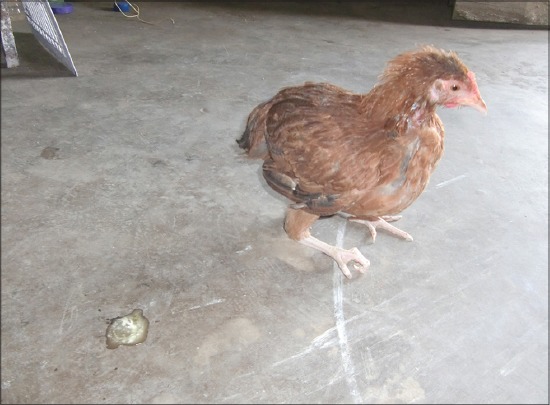
Chicken with curled toes.

The acute toxicity group of chickens developed signs of toxicity 2 h after dosing, which included excitation followed by sluggishness, greenish bloody diarrhea (Figure-1), excessive salivation, progressing to drooling, and rigid stance with drooping of wings. Progressively the birds were unable to stand and sat on their hocks with curled toes (Figure-2) followed by tremor, convulsions and recumbency before death. Finally, all the chickens died within 4-36 h of dosing. Signs of toxicity were evident within 2 h following oral administration of CPF. This might be due to rapid absorption, distribution, and metabolism profile of CPF. The main action of OP insecticides in the avian species, as well as in mammals, is inhibition of cholinesterase (CHE) activity with subsequent development of cholinergic overstimulation manifested as nicotinic, muscarinic, and central nervous effects. Muscarinic symptoms such as diarrhea and salivation, nicotinic symptoms such as muscle tremors, paresis progressing to paralysis, central nervous effects like seizures are due to reversible blockade of acetyl CHE, with consequent accumulation of acetylcholine which causes excessive synaptic neurotransmitter activity in the parasympathetic (cholinergic) nervous system at neuromuscular (cholinergic) site [6].

The Hb, TEC, and TLC concentration of acute toxicity group at every 6 h interval up to 36 h, after administration of CPF are presented in Table-1. The Hb, TEC, and TLC concentration was significantly (p<0.05) increased from 6 h onward as compared to control chickens. The increased Hb concentrations recorded in the present study might be due to severe diarrhea causing dehydration resulting in hemoconcentration. This observation was in agreement with the findings of earlier workers [7-9]. The increase in TEC might be due to dehydration resulting from diarrhea and excessive salivation. Similar observation was also recorded in chickens [9]. The increase in TLC could be due to dehydration leading to hemoconcentration, which simulates the findings of Malik *et al.*, [9]. Further, it was reported that intoxication caused leukocytosis [7]. Dose-dependent significant increase of TLC was observed in rats exposed to high dose of CPF [10]. The mean values of lymphocyte and heterophil percent in treated and control chickens are presented in Table-1. In the treated group, the lymphocyte percent showed decreasing trend than the control group. The statistical analysis revealed that there was significant (p<0.01) variation of lymphocyte percent from 6 h onward between control and treated groups. The mean values of heterophil percent in treated and control chickens are presented in Table-1. In the treated group, the heterophil percent showed increasing trend than the control group. The statistical analysis revealed that there was significant (p<0.01) increase of heterophil percent from 6 h onward between control and treated groups. The significant increase in heterophil percent and decrease in lymphocyte percent might be due to acute degenerative changes in various organs as observed under microscope leading to increase heterophil population and the resultant decrease in lymphocyte percent. The lymphopenia observed in the present study might be due to systemic stress caused by CPF toxicity. It was reported that almost all chemical toxicants increase glucocorticoid level in the blood due to stress resulting in heterophilia [11]. There was no variation in the monocyte, eosinophil, and basophil percent in the treated groups compared to control groups.

**Table-1:** Effect of CPF on hematology (mean±SE) in indigenous chicken.

Hematology	Control					CPF				
	Time of blood collection (h)					Time of blood collection (h)				
	
	0	6	12	24	36	0	6	12	24	36
Hb (g %)	10.67±0.39	10.73±0.37	10.77±0.40	10.77±0.40	11.00±0.41	9.92±0.36	12.35±0.39*	13.10±0.24**	13.00±0.20*	13.30±0.30*
TEC (10^6^/µl)	2.31±0.14	2.42±0.14	2.51±0.15	2.52±0.08	2.63±0.16	2.34±0.15	2.96±0.21*	3.25±0.16 **	2.52±0.08	3.99±0.01**
TLC (10^3^/µl)	24.53±1.35	24.56±1.35	24.66±1.35	24.56±1.35	24.57±1.34	25.04±1.44	28.07±0.81*	33.13±0.38*	29.74±0.75**	31.21±0.84**
Lymphocyte (%)	54.16±1.30	54.16±1.38	54.08±1.63	54.50±1.95	54.33±1.43	52.5±1.03	38.50±2.79**	42.00±1.04**	41.00±1.41**	41.50±0.35**
Heterophil (%)	32.83±1.45	32.33±1.31	32.83±1.58	32.66±1.82	32.66±1.52	34.67 ±1.41	42.00±1.47**	44.50±1.26**	45.50±1.50**	46.50±1.50**

CPF=Chlorpyriphos, SE=Standard error

The mean ALP, AST, ALT, CHE activities and level of total protein and uric acid at different hours in control and treated chickens have been presented in Table-2. The mean ALP, AST, and ALT activities increased significantly (p<0.05) from 6 h onward till the end of the experiment. The mean values of CHE activity showed significant (p<0.05) inhibition from 6 h to 36 h between treated and control groups. In the present investigation, the CPF treated chickens showed dose-dependent increase of ALP, AST, and ALT activities with significantly (p<0.05) increased values up to 36 h of observation in acute toxicity group chickens. The increase level of AST and ALT might be due to degeneration and necrosis of hepatocytes. Increase of plasma AST and ALT activity is the most specific indicator of muscle and liver cell damage [12]. Increased enzyme concentrations are a measure of recent organ damage rather than decreased organ function, [12] which was in agreement with the present observation. The decreased CHE activity observed in the present study could be due to binding of CPF to CHE enzyme. Reduced CHE activity is a reliable indicator of OP poisoning and a biomarker of absorption of OP insecticides [6]. In the present study, the level of CHE was found to be reduced significantly in acute toxicity group. The significant reduction of plasma CHE activity in CPF treated group in the present study is supported by earlier reports of CPF toxicity in broiler chicks [13]. Banaee *et al.*, 2011 reported that the release of intercellular enzymes into the blood and their increased activity in plasma are the most important clinical signs in the diagnosis of damage to cell membranes [14]. Akhtar *et al.*, 2009 reported significant dose-dependent inhibition of blood acetyl CHE activity in rats [10].

**Table-2:** Effect of CPF on serum biochemical parameters (mean±SE) in indigenous chicken.

Biochemical parameters	Control					CPF				
	Time of blood collection (h)					Time of blood collection (h)				
	
	0	6	12	24	36	0	6	12	24	36
ALP (IU/L)	731.81±3.39	731.82±3.39	731.81±3.39	731.81±3.39	731.83±3.40	601.52±1.99	842.58±1.35*	976.45±1.78*	1100.39±1.01*	1147.99±1.98*
AST(IU/L)	343.38±2.47	343.38±2.47	343.39±2.47	343.36±2.47	343.39±2.47	278.53±1.37	599.09±3.46*	729.37±2.80*	810.52±3.45*	920.89±3.75*
ALT (IU/L))	3.96±0.19	3.96±0.19	3.95±0.19	3.95±0.19	3.97±0.19	3.18±0.31	6.82±0.66*	9.75±0.48*	11.49±0.04*	21.89±0.81*
CHE (IU/L)	786.37±1.25	786.36±1.25	786.37±1.25	786.36±1.25	786.37±1.25	839.92±2.79	547.97±3.90*	381.42±3.23*	413.03±3.19*	307.35±3.70*
Total protein (g/dl) (%)	3.85±0.28	3.87±0.28	3.86±0.28	4.00±0.28	4.05±0.27	3.59±0.23	3.79±0.31	3.78±0.31	3.85±0.10	3.89±0.11
Uric acid (mg/dl)	3.81±0.46	3.81±0.45	3.81±0.46	3.81±0.46	3.81±0.45	3.53±0.25	8.80±0.59*	12.71±0.18*	12.55±0.30*	15.28±0.38*

* Mean in a column bearing same subscript and mean in a row bearing same superscript do not differ significantly (p<0.05), (n=6, mean±SE),

SE=Standard error, ALP=Alkaline phosphatase, AST=Aspartate aminotransferase, ALT=Alanine aminotransferase, CHE=Cholinesterase, CPF=Chlorpyriphos

The mean serum total protein in control and a treated group of chickens are presented in Table-2. The analysis of the data on total protein showed variation between control and treated groups. The analysis of the data on total protein showed variation between control and treated groups, but it was not significant which was in agreement with earlier works of Kammon *et al*. [13].

The mean serum uric acid in control and treated Group are presented in Table-2. Analysis of the activities between the groups showed significant (p<0.05) increase from 6 h to 36 h between treated and control groups. Significant increase in uric acid level observed in the present study might be due to renal tubular necrosis, seen in the histopathological examination. Transient renal injury in OP poisoning causes a subsequent increase in the uric acid level [13].

## Conclusion

From the present investigation, it can be inferred that exposure to CPF produces hematological and biochemical alterations in indigenous chicken. Thus, based on the results of the present findings it is established that CPF produces hematological and biochemical, alterations in indigenous chicken. However, the exact mechanism that caused cell damage leading to hematological and biochemical alterations needs to be elucidated.

## Authors’ Contributions

SAB and TNU designed and planned the study. SAB done the experiment and drafted the manuscript. GKB, TR, DCP and KS assisted in conduct of the study and revision of the manuscript. RSB done the statistical analysis. All authors read and approved the final manuscript.
